# High Performance Near-Infrared Emitters with Methylated Triphenylamine and Thiadiazolo[3,4-g]quinoxaline-Based Fluorophores

**DOI:** 10.3390/molecules26216386

**Published:** 2021-10-22

**Authors:** Youming Zhang, Chengjun Wu, Minrong Zhu, Jingsheng Miao

**Affiliations:** Shenzhen Key Laboratory of Polymer Science and Technology, College of Materials Science and Engineering, Shenzhen University, Shenzhen 518060, China; zhangym@szu.edu.cn (Y.Z.); 2070343051@email.szu.edu.cn (C.W.); zhumr@szu.edu.cn (M.Z.)

**Keywords:** near-infrared emitters, hybridized local and charge transfer, organic light-emitting diodes, electroluminescence

## Abstract

Three near-infrared emitters (**2TPA-QBT**, **2MeTPA-BT** and **TPA-QBT-MeTPA**) were rationally designed and synthesized. Density functional theory (DFT) and time-dependent density functional theory (TDDFT) calculations showed that the introduction of mono- or di-methyl groups between the donors and acceptor could result in the spatial configuration changing greatly for **2MeTPA-QBT** and **TPA-QBT-MeTPA** compared to their parent compound **2TPA-QBT**. The emission of **TPA-QBT-MeTPA** had a more obvious hybridized local and charge transfer feature (HLCT) based on the influence of the steric hindrance of the methyl substituent. Attributed to their different spatial configurations and luminescence mechanisms, different emission wavelengths with photoluminescent quantum yields of 26%, 38% and 34% in toluene, as well as 24%, 27% and 31% in 4,4′-bis(N-carbazolyl)-1,1′-biphenyl (CBP) doped film, were observed for **2TPA-QBT**, **2MeTPA-QBT** and **TPA-QBT-MeTPA**, respectively. The constructed organic light-emitting devices (OLEDs) displayed electroluminescence with emission peaks at 728, 693 and 710 nm, with maximum external quantum efficiencies of 1.58%, 1.33% and 3.02% for the **2TPA-QBT**, **2MeTPA-QBT** and **TPA-QBT-MeTPA**-doped OLEDs, respectively. This work illustrated the effect of spatial configuration changes on the luminescence properties of donor-acceptor-type organic emitters.

## 1. Introduction

The tuning of the emission wavelength of organic light-emitting diodes (OLEDs) to the deep-red/near-infrared (DR/NIR) region has attracted widespread attention since DR/NIR light has a wide range of applications in night-vision displays, sensors and optical communications, as well as offering superior biocompatibility for medical systems [1,2,3,4,5]. To achieve this, tremendous efforts are devoted to achieving DR/NIR emission in the range of 650–900 nm from transition-metal complexes, boron dipyrromethene dyes and fluorescent materials with donor-acceptor (D-A) structures [5,6,7,8]. According to spin quantum theory, the ratio of the singlet to triplet exciton is 1:3 for exciton recombination process. Therefore, the utilization of the triplet exciton energy is the essential factor to obtain highly efficient devices [9,10,11]. Based on this, transition-metal complexes have attracted significant attention because they can provide a strong spin-orbit coupling between the triplet state and ground state to achieve 100% internal quantum efficiency [12,13,14,15,16]. Such devices based on transition-metal complexes can usually achieve high luminescence efficiency [17,18,19]. However, the sensitivity to oxygen and moisture, as well as the environmental toxicity and high costs, could limit the application and development of metal complexes-based OLEDs. Because of this, metal-free organic materials have attracted considerable research interest due to the advantages of their short exciton lifetime, tunable band gaps, molecular structure diversity, low costs, as well as their capability for batch production [20,21,22,23]. 

In the last decade, for utilizing both singlet and triplet excitons, hybridized local and charge-transfer state (HLCT) and thermally activated delayed fluorescence (TADF) materials was proposed by controlling the excited state and regulating the orbital separation between the highest occupied molecular orbital (HOMO) and the lowest unoccupied molecular orbital (LUMO), and break the 5% external quantum efficiency (EQE) limit in OLEDs [20,22,24,25,26,27,28]. For HLCT emission, the reverse intersystem crossing (RISC) of excitons from the triplet state (T_n_) to singlet state (S_n_) with close energy levels can also occur in the excited state. This state combines both local excitation (LE) and charge transfer (CT) states into a special one that possesses two combined and compatible characteristics: a large transition moment from the LE state (cold exciton) and a weakly bound exciton from the CT state (‘‘hot’’ exciton). For TADF, a smaller value of the S_1_-T_1_ band gap (∆*E*_ST_) is beneficial for harvesting triplet excitons via reverse intersystem crossing (RISC). The value of ∆*E*_ST_ is related to the overlap integral of the HOMO and the LUMO. DR/NIR emission materials based HLCT/TADF characteristics were also reported. For example, Liu et al. reported a D-π-A-π-D-type NIR emitter of NZ2TPA, which showed HLCT features and an EQE of 3.9% with an emission peak at 696 nm in undoped electroluminescent device [29]. Li et al. reported a D-A-type DR/NIR emitter of TPA-QCN, with TADF properties and high EQEs of 3.9–14.5% when doped under different concentrations in OLED devices [30].

**Figure 1:**
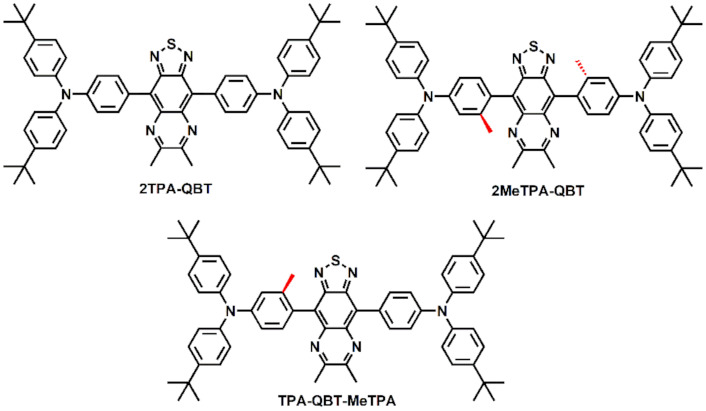
Chemical structures of emitters.

In a previous study, we synthesized emitters with HLCT emission features by introducing methyl groups into small symmetric molecules [31]. The results showed that the introduction of a single methyl group (2MeTPA-BT) made the HLCT properties more obvious. Herein, in order to obtain NIR electroluminescent materials, a [1,2,5]thiadiazolo[3,4-*g*]quinoxaline acceptor, which had a stronger electron-withdrawing ability and larger conjugation, was selected to replace benzo[c] [1,2,5]thiadiazole. A D-A-D/D’-type emitter of **2MeTPA-QBT** was synthesized by introducing a single methyl group into each D unit. In addition, an asymmetric emitter, **TPA-QBT-MeTPA**, was obtained by introducing single methyl group into one of the D units. Their parent compound **2TPA-QBT** was also synthesized for comparison (Figure 1). Due to steric hindrance, the torsional angles between the D and A units were different. Their optimized configuration torsional angles between the D and A units of ~44°, ~ 61° and ~ 44°/~61° were observed for **2TPA-QBT**, **2MeTPA-QBT** and **TPA-QBT-MeTPA**, respectively. Density functional theory (DFT) analysis revealed that the emission of **TPA-QBT-MeTPA** had a more obvious HLCT feature. The doped OLEDs based on **TPA-QBT-MeTPA** achieved a maximum EQE of 3.02% with an emission peak at 707 nm and luminance of 1875 cd m^−2^. 

## 2. Results and Discussion

### 2.1. Synthesis and Thermal Stability

**Figure 2:**
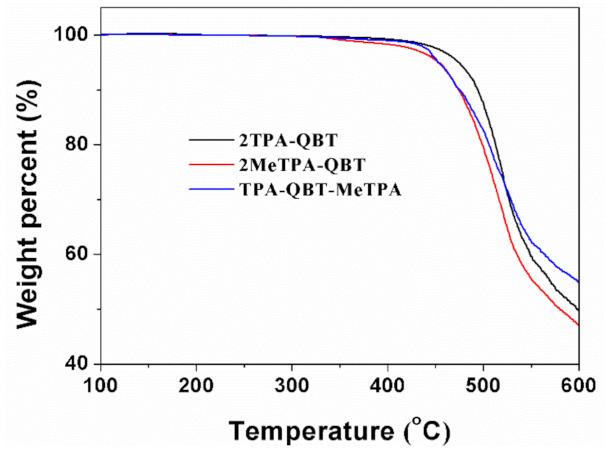
Thermogravimetric analysis curves for **2TPA-QBT**, **2MeTPA-QBT** and **TPA-QBT-MeTPA**.

The starting materials and solvents are purchased available and used as received. All operations involving oxygen-sensitive reagents were performed under argon. **MeTPA-Br**, **TPA-Bpin** and **MeTPA-Bpin** were synthesized according the reported reference by Pd-catalyzed C−N cross-coupling reactions [31]. **QBT-2Br** was synthesized by amine-aldehyde condensation [32]. The final products of **2TPA-QBT, 2MeTPA-QBT** and **TPA-QBT-MeTPA** were prepared using the Suzuki coupling reaction using Pd(PPh_3_)_4_ as a catalyst. All the new compounds were characterized using NMR spectroscopy and mass spectrometry, confirming their well-defined chemical structures. Detailed synthesis processes are provided in the supporting information (Supplementary Material, Figure S1). Figure 2 shows the thermogravimetric analysis curves of obtained emitters under a N_2_ atmosphere. The decomposition temperatures (*T*_d_) with 5% weight loss were found to be 473, 453 and 453 °C for **2TPA-QBT, 2MeTPA-QBT** and **TPA-QBT-MeTPA**, respectively, which revealed that these organic emitters had enough high thermal stability for the fabrication of OLEDs.

### 2.2. Electrochemical Properties

**Figure 3:**
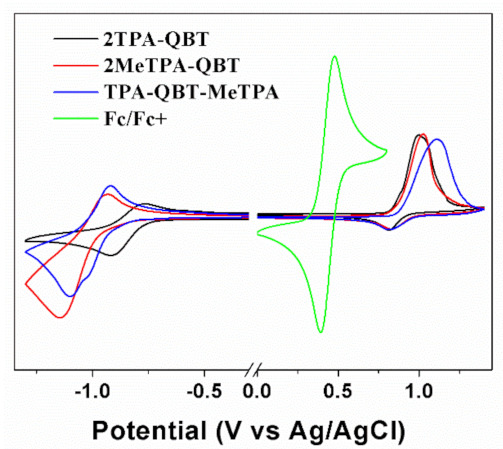
Cyclic voltammograms for emitter films on a platinum electrode in an acetonitrile solution containing 0.1 mol L^−1^ Bu_4_NPF_6_ at a 100 mV s^−1^ scan rate using a ferrocene internal standard.

As depicted in Figure 3, cyclic voltammetry curves for neat films of **2TPA-QBT**, **2MeTPA-QBT** and **TPA-QBT-MeTPA** coated on a platinum electrode were measured. The calculating formula of *E*_HOMO_ = [−(*E*_ox_ − 0.44) − 4.8] eV and *E*_LUMO_ = [−(*E*_red_ − 0.44) − 4.8] eV were used to estimate the HOMO (*E*_HOMO_) and LUMO energy levels (*E*_LUMO_) by the onset oxidation/reduction potentials (*E*_ox_/*E*_red_), where 0.44 V is the potential of ferrocene relative to Ag/AgCl and 4.8 eV is the energy level of ferrocene with respect to the vacuum energy level [33]. Three approximate reversible redox couples of 0.81/−0.73, 0.82/−0.88 and 0.85/−0.80 V versus Ag/AgCl were found for **2TPA-QBT**, **2MeTPA-QBT** and **TPA-QBT-MeTPA** (Table 1), respectively, which were attributed to the first oxidation of the donor units and the first reduction of the acceptor unit [31]. Compared to **2TPA-QBT**, the oxidation potentials of the emitters increased slightly and the reduction potentials decreased more obviously for both **2MeTPA-QBT** and **TPA-QBT-MeTPA**. Ultimately, this led to a gradual increase in the electrochemical band gaps ($E_{cv}^{g}$) of 1.54, 1.70 and 1.65 eV for **2TPA-QBT**, **2MeTPA-QBT** and **TPA-QBT-MeTPA**, respectively. 

### 2.3. Photophysical Properties

**Figure 4:**
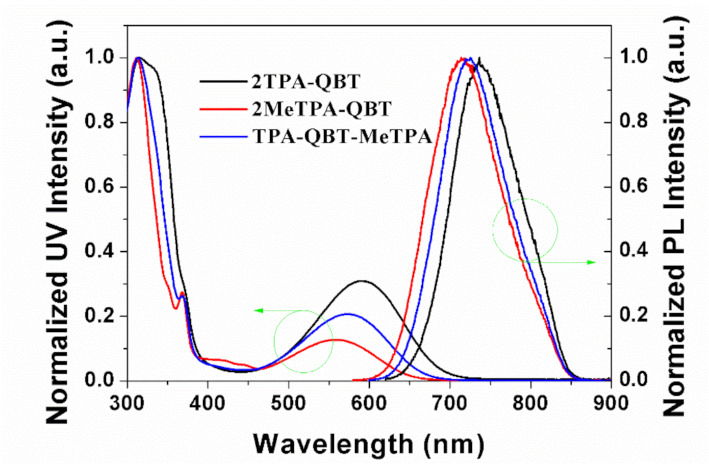
Ultraviolet-visible absorption and photoluminescence spectra for **2TPA-QBT**, **2MeTPA-QBT** and **TPA-QBT-MeTPA** in toluene solution.

Figure 4 shows the UV-vis absorption and fluorescence spectra of emitters in toluene. The intense absorptions at <350 nm were assigned to the intramolecular π-π* electronic transition. The broad absorptions at 500–700 nm were attributed to intramolecular charge transfer (ICT) transitions from D to A units. Almost the same π-π* electronic absorptions occurred at ~310 nm for all three emitters, but different ICT absorption bands were observed (589, 557 and 574 nm for **2TPA-QBT**, **2MeTPA-QBT** and **TPA-BT-MeTPA**, respectively). Due to the increase of torsion in molecular space, the intensity of the ICT absorption bands gradually decreased in the order of **2TPA-QBT** > **TPA-QBT-MeTPA** > **2MeTPA-QBT**. This suggested that the intramolecular interactions between D and A units correspondingly decreased which were induced by the introduced methyl groups. Moreover, these emitters exhibited similar absorption curves and the absorption peak only changed minimally with the solvent polarities changed, implying a rather small dipolar change in the ground state for different solvents (Supplementary Material, Figures S2–S4) [34,35,36]. Interestingly, an obvious narrow absorption peak at 368 nm was observed for **2MeTPA-QBT** and **TPA-BT-MeTPA**. This may be induced by the hindered rotation between D and A units due to the introduction of methyl groups. Red-shifted absorption spectra were obtained in the neat films due to their stronger intermolecular interaction compared to solution state (Supplementary Material, Figure S5). Based on the onsets of film absorption, we calculated the optical band gaps ($E_{g}^{opt}$) as 1.62, 1.72 and 1.68 eV for **2TPA-QBT**, **2MeTPA-QBT** and **TPA-QBT-MeTPA**, respectively. 

The photoluminescence (PL) spectra in different solvents for emitters are depicted in Supplementary Material, Figures S6–S8. Different from the absorption properties, broadened PL spectra with a remarkable redshift were observed as the solvent polarity increased. The emission peaks shifted from 714, 662 and 694 nm in nonpolar hexane to 780, 796 and 788 nm in polar dichloromethane for **2TPA-QBT**, **2MeTPA-QBT** and **TPA-QBT-MeTPA**, respectively. The large solvatochromic shift indicated a strong interaction between D and A unit. Furthermore, the neat film of emitters produced emission peaks at 780, 732 and 748 nm for **2TPA-QBT**, **2MeTPA-QBT** and **TPA-QBT-MeTPA**, respectively (Figures S9–S11). Correspondingly, the PLQYs of the emitters in toluene were 26%, 38% and 34%, and in neat films were 5%, 6% and 8% for **2TPA-QBT**, **2MeTPA-QBT** and **TPA-QBT-MeTPA**, respectively. However, the PLQYs increased significantly when blended in 4,4′-bis(N-carbazolyl)-1,1-biphenyl (CBP) films of 24%, 27% and 31% compared to in neat films for **2TPA-QBT**, **2MeTPA-QBT** and **TPA-QBT-MeTPA**, respectively. The lowered PLQYs in the neat films indicated that these emitters suffered from aggregation-caused quenching (ACQ) due to molecular stacking. Only nanosecond level emission lifetime in the neat film was observed, and the exciton lifetime hardly changes at different temperatures. This is quite different from the TADF and phosphorescence materials, which suggesting that there was no RISC process from the lowest triplet state (T_1_) to the lowest singlet state (S_1_) (Supplementary Material, Figures S12–S14). The resulting photophysical data are summarized in Table 2. 

**Figure 5:**
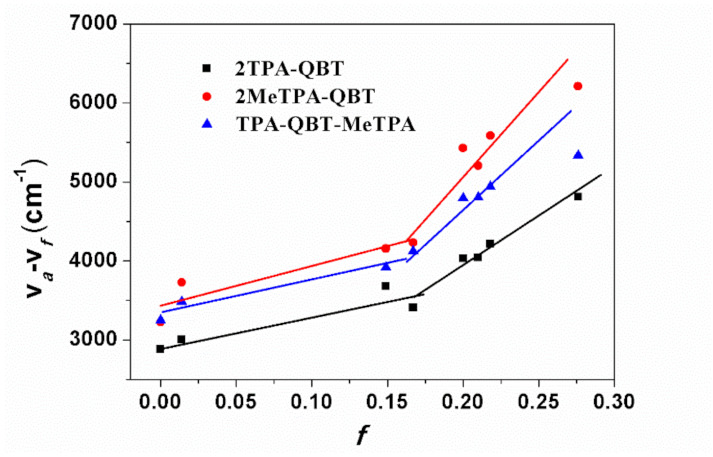
Linear correlation of orientation polarity *f* of solvent media with the Stokes shift (v*_a_*-v*_f_*) for all emitters.

Lippert–Mataga expression was used for better understanding the relationship between the Stokes shift and solvent polarity parameter (*f*) to evaluate the ICT effect [37,38,39]. As depicted in Figure 5, two-stage linear relations were simulated in respective low and high polarity solvents for all emitters. The dipole moments (*μ*_e_) of 12.6, 13.8 and 11.6 D in low polarity solvents, as well as 29.8, 31.5 and 30.7 D in high polarity solvents, were calculated for **2TPA-QBT**, **2MeTPA-QBT** and **TPA-QBT-MeTPA**, respectively. Similar to earlier studies, the smaller *μ*_e_ in less polar solvents was attributed to the LE state, while the larger *μ*_e_ in highly polar solvents was assigned to the CT excited state. The HLCT state was formed through the coupling and intercrossing between the CT and LE states. More detailed photophysical data are summarized in Table S1 (Supplementary Material).

### 2.4. Theoretical Calculations

**Figure 6:**
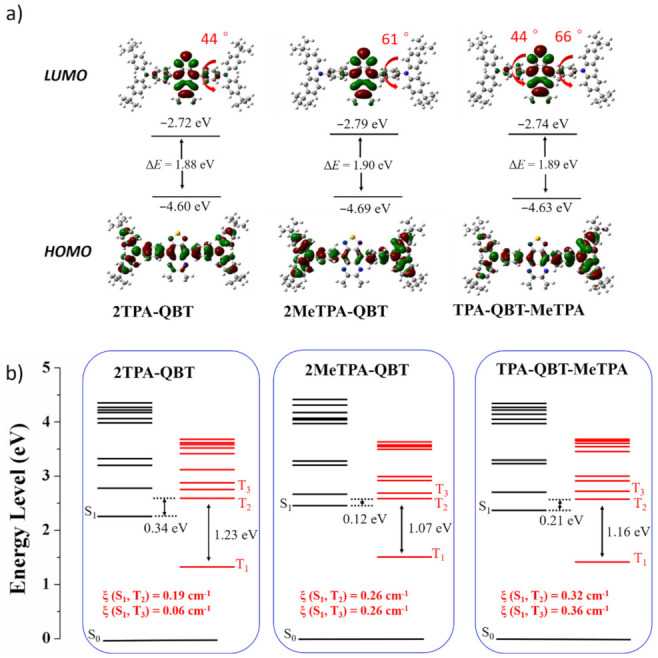
(**a**) Optimized structure and calculated HOMO/LUMO spatial distributions and (**b**) energy level diagrams of emitters.

Quantum calculations were performed using the Gaussian 09 suite of programs at the B3LYP/6-31G(d) level for further understanding their photophysical properties [40]. Due to steric hindrance of methyl, different torsional angles of ~44° for **2TPA-QBT**, ~61° for **2MeTPA-QBT** and ~44°/~61° for **TPA-QBT-MeTPA** between the D and A units were observed in their optimized configuration, respectively (Figure 6a). As expected, the HOMO distributions were mainly located on the D units and the LUMO levels were mostly distributed on the A unit. Furthermore, with the torsion increased, the LUMO were gradually centralized toward the central A unit, while the HOMO were more dispersed on the D units. Thus, the introduced methyl effectively regulate the overlap of HOMO and LUMO orbitals.

The natural transition orbitals (NTOs) were also further calculated to analyze the electron transition characters (Supplementary Material, Figures S15–S17). For the singlet states (S_1_) of **2TPA-QBT**, the holes were located on the whole molecule and the particles were centralized on the central A component, suggesting the existence of both CT and LE states. In contrast, for the singlet states (S_1_ and S_2_) of **2MeTPA-QBT** and **TPA-QBT-MeTPA**, the holes and particles were centralized on the D and A components, respectively, implying that there was only the CT state. For the first-excited triplet state (T_1_) of all emitters, the overlap of the distribution for holes and particles demonstrated the coexistence of CT and LE components. However, for the second-excited triplet state (T_2_), only the CT state was observed for all emitters. 

There were large band gaps of 1.23, 1.07 and 1.16 eV between T_2_ and T_1_, as well as band gaps of 0.94, 0.95 and 0.95 eV between S_1_ and T_1_ for **2TPA-QBT**, **2MeTPA-QBT** and **TPA-QBT-MeTPA**, respectively. These large band gaps may suppress the internal conversion rate from T_2_ to T_1_ and the RISC rate of T_1_–S_1_ according to the band-gap law. However, small band gaps between S_1_ (and/or S_2_) and T_2_ (or T_3_) were obtained for all emitters, which could facilitate the RISC process from T_2_ (and/or T_3_) to S_1_ (and/or S_2_). These characteristics of NTOs and excited energy levels were in accordance with the ‘‘hot’’ exciton principle, thereby leading to high production efficiency of radiative singlet excitons (***η***_s_) for these emitters. The computed spin-orbit coupling (SOC) matrix element values of **ξ** (S_1_, T_2_)/**ξ** (S_1_, T_3_) were 0.19/0.06, 0.26/0.26 and 0.32/0.36 cm^−1^ for **2TPA-QBT**, **2MeTPA-QBT** and **TPA-QBT-MeTPA**, respectively. The larger value of SOC may lead to a more enhanced RISC efficiency for **TPA-QBT-MeTPA**. 

### 2.5. Electroluminescence Properties

**Figure 7:**
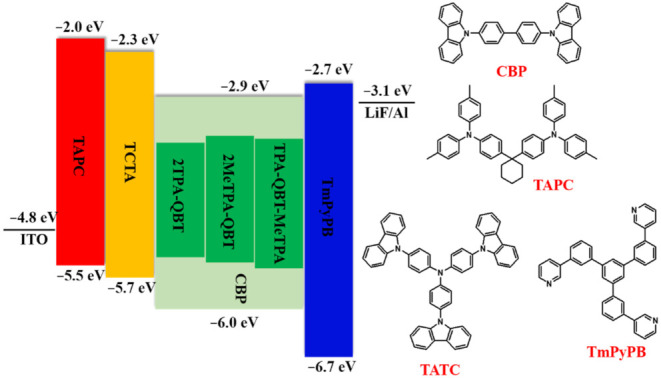
Energy diagram of OLEDs and the chemical structures of adopted materials.

To research their electroluminescence (EL) properties, OLEDs were fabricated by using these emitters as dopants with the configuration of indium tin oxide (ITO)/1,1-bis[4-[*N*,*N*′-di(p-tolyl)amino]-phenyl]-cyclohexane (TAPC, 30 nm)/4,4′,4″-tris(carbaz;tris(4-(9*H*-carbazol-9-yl) (TCTA, 15 nm)/emitter: 4,4′-bis(*N*-carbazolyl)-1,1′-biphenyl (CBP, 5 wt.%, 15 nm)/1,3,5-tri[(3-pyridyl)phen-3-yl]benzene (TmPyPB, 65 nm)/LiF (1 nm)/Al (100 nm), where CBP, TAPC, TCTA and TmPyPB served as the host material, hole injection, hole transporting and electron transporting layers, respectively (Figure 7). Emitters were doped into the CBP host with an optimal doping concentration of 5 wt.% to serve as the EMLs. 

**Figure 8:**
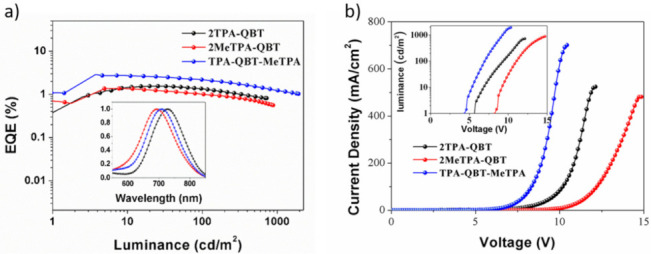
(**a**) External quantum efficiency-luminance curves. Inset: normalized EL spectra of the devices. (**b**) Current density-voltage-luminance curves.

As shown in Figure 8, the devices exhibited emission peaks at 718, 693 and 707 nm for the **2TPA-QBT-**, **2MeTPA-QBT-** and **TPA-QBT-MeTPA**-based OLEDs, respectively. Compared to **2TPA-QBT**, the EL peaks of the **2MeTPA-QBT-** and **TPA-QBT-MeTPA**-based OLEDs showed obvious blue shifts of 25 and 11 nm from the PL maxima for the corresponding neat films. The devices exhibited maximum external quantum efficiencies (EQE_max_) of 1.58%, 1.33% and 3.02% for the **2TPA-QBT-**, **2MeTPA-QBT-** and **TPA-QBT-MeTPA**-doped OLEDs with Commission Internationalede L’Eclairage (CIE) coordinates of (0.70, 0.31), (0.70, 0.29) and (0.69, 0.30), turn-on voltages (*V*_on_) of 5.6, 8.3 and 4.4 V and maximum luminance values (*L*_max_) of 783, 839 and 1875 cd m^−2^ for **2TPA-QBT-**, **2MeTPA-QBT-** and **TPA-QBT-MeTPA**-based OLEDs, respectively. Furthermore, the PLQYs of the active layer composed of emitter:CBP (5 wt.%) were measured, with values of 24%, 27% and 31% obtained for the **2TPA-QBT-**, **2MeTPA-QBT-** and **TPA-QBT-MeTPA**-doped films, respectively. The EL data for all the emitters are summarized in Table 3.

## 3. Conclusions

To summarize, three D-A-D-type NIR emitters (**2TPA-QBT**, **2MeTPA-QBT** and **TPA-QBT-MeTPA**) were designed and synthesized. Introducing methyl steric hindrance between the D and A units can cause different torsional angles, and further regulate the overlap of HOMO and LUMO orbitals. The emission of the emitters proved to be governed by an HLCT mechanism. These emitters showed different emission wavelengths with acceptable PLQYs in the toluene and neat film. OLEDs using these emitters as single dopant exhibited NIR emission with EL peaks at 718, 693 and 707 nm and maximum EQEs of 1.58%, 1.33% and 3.02% for **2TPA-QBT-**, **2MeTPA-QBT-** and **TPA**-**QBT**-**MeTPA**-doped OLEDs, respectively. This work showed the effect of spatial configuration changes on the luminescence properties of NIR organic emitters.

## Figures and Tables

**Table 1:** Thermal stability and electrochemical data for the three emitters.

Emitters	*E*_ox_^a^/V	*E*_red_^a^/V	*E*_HOMO_^b^/eV	*E*_LUMO_^b^/eV	*E*_cv_/eV	*T*_d_/°C
**2TPA-QBT**	0.81	−0.73	−5.17	−3.63	1.54	473
**2MeTPA-QBT**	0.82	−0.88	−5.18	−3.48	1.70	453
**TPA-QBT-MeTPA**	0.85	−0.80	−5.21	−3.56	1.65	453

^a^ Onset oxidation and reduction potentials measured by cyclic voltammetry in solid films. ^b^ *E*_HOMO_ = [−(*E*_ox_ − 0.44) − 4.8] eV, *E*_LUMO_ = [−(*E*_red_ − 0.44) − 4.8] eV, where 0.44 V is the value for ferrocene vs. Ag/AgCl and 4.8 eV is the energy level of ferrocene relative to the vacuum energy level.

**Table 2:** Photophysical properties for the three emitters.

Emitters	λ_abs sol_/nm ^a^	λ_abs film/_nm ^b^	λ_em sol_/nm ^a^	λ_em film_/nm ^b^	$E_{g}^{opt}$/eV ^c^	Φ_F_/% _sol_^d^	τ_sol_/ns
**2TPA-QBT**	589, 314	609	736	780	1.62	26	6^a^/3^b^
**2MeTPA-QBT**	557, 312	574	714	732	1.72	38	5^a^/6^b^
**TPA-QBT-MeTPA**	557, 312	585	724	748	1.68	34	7^a^/3^b^

^a^ Measured in toluene (10^−5^ mol L^−1^); ^b^ measured in neat film; ^c^ optical band gaps were determined using $E_{g}^{opt}$_film_ = 1240/λ_onset, film_; ^d^ absolute PL quantum yield measured using an integrating sphere.

**Table 3:** Electroluminescence parameters for the three doped devices.

Emitters	*λ*_EL_ (nm) ^a^	V_on_ (V) ^b^	EQE (%) ^c^	*L*_max_ (cd/m^2^) ^d^	CIE ^e^
**2TPA-QBT**	718	5.6	1.58	783	(0.70, 0.31)
**2MeTPA-QBT**	693	8.3	1.33	839	(0.70, 0.29)
**TPA-QBT-MeTPA**	707	4.4	3.02	1875	(0.69, 0.30)

^a^ Maximum EL emission peak; ^b^ turn-on voltage; ^c^ maximum external quantum efficiency; ^d^ maximum luminance; ^e^ color coordinates.

## Data Availability

Data on the compounds are available from the authors.

## Supplementary Materials

The supplementary materials are available online. Figure S1. Synthetic routes of **2TPA-QBT, 2MeTPA-QBT** and **TPA-QBT-MeTPA**. Figure S2. UV-vis absorption spectra of **2TPA-QBT** in different solvents. Figure S3. UV-vis absorption spectra of **2MeTPA-QBT** in different solvents. Figure S4. UV-vis absorption spectra of **TPA-QBT-MeTPA** in different solvents. Figure S5. UV–vis absorption spectra of emitters in neat films. Figure S6. Photoluminescence spectra of **2TPA-QBT** in different solvents. Figure S7. Photoluminescence spectra of **2MeTPA-QBT** in different solvents. Figure S8. Photoluminescence spectra of **TPA-QBT-MeTPA** in different solvents. Figure S9. Variable-temperaturephotoluminescence spectra of **2TPA-QBT** in neat films. Figure S10. Variable-temperaturephotoluminescence spectra of **2MeTPA-QBT** in neat films. Figure S11. Variable-temperaturephotoluminescence spectra of **TPA-QBT-MeTPA** in neat films. Figure S12. Variable-temperaturephotoluminescence decay characteristics of **TPA-QBT-MeTPA** in neat films. Figure S13. Variable-temperaturephotoluminescence decay characteristics of **2MeTPA-QBT** in neat films. Figure S14. Variable-temperaturephotoluminescence decay characteristics of **TPA-QBT-MeTPA** in neatfilms. Figure S15. The energy level and the natural transitionorbitals of **2TPA-QBT**. Figure S16. The energy level and the natural transitionorbitals of **2MeTPA-QBT**. Figure S17. The energy level and the natural transitionorbitals of **TPA-QBTMeTPA**. Table S1. Photophysical properties for the three emitters.

## Sample Availability

Samples of the compounds are available from the authors.
