# Supplementary material for: High Performance Near-Infrared Emitters with Methylated Triphenylamine and Thiadiazolo[3,4-g]quinoxaline-Based Fluorophores
Source: Molecules. 2021 Oct 22;26(21):6386. doi: 10.3390/molecules26216386 (PMC8588353; doi:10.3390/molecules26216386)

High performance near-infrared emitters with methylated triphenylamine and thiadiazolo[3,4-g]quinoxaline-based fluorophores

Youming Zhang, Chengjun Wu, Minrong Zhu, and Jingsheng Miao*

Shenzhen Key Laboratory of Polymer Science and Technology, College of Materials Science and Engineering, Shenzhen University, Shenzhen 518060, China

*Correspondence: jingshengmiao@szu.edu.cn

1. General considerations for characterization

Instrument: Nuclear magnetic resonance (NMR) spectra were recorded on a Bruker DRX 400 spectrometer using tetramethylsilane as a reference in deuterated chloroform solution at 298 K. MALDI-TOF mass spectrometric measurements were performed on a Bruker Bifiex III MALDI-TOF spectrometer. Thermogravimetric analysis (TGA) was conducted under a dry nitrogen gas flow at a heating rate of 10 ^{o}C min^{-1} on a Perkin- Elmer TGA 7. Cyclic voltammetry was carried out on a CHI660A electrochemical work station in a three-electrode cell dipped in a 0.1 M tetrabutylammonium hexafluorophosphate (Bu_4NPF_6) acetonitrile solution under nitrogen protection at a scan rate of 100 mV s^{-1} and room temperature (RT). In this three-electrode cell, a platinum rod, platinum wire and Ag/AgCl electrode were used as a working electrode, counter electrode and reference electrode, respectively. Ferrocene was added as the internal standard. Shimadzu UV-2700 spectrophotometer (Shimadzu, Japan) was applied to record UV-vis spectra at 25 ^{o}C. Photoluminescence (PL) spectra in different solvents were determined on a Hitachi F-7100 fluorescence spectrophotometer (Hitachi, Japan) at 25 ^{o}C. The variable-temperature PL spectura and variable-temperature transient PL decay curves in neat film were obtained by FluoTime 300 (PicoQuant GmbH) with a Picosecond Pulsed UV-LASTER (LASTER485) as the excitation source. The photoluminescence quantum yields (*PLQYs*) were achieved by a Hamamatsu UV-NIR absolute PL quantum yield spectrometer (C13534, Hamamatsu Photonics) equipped with a calibrated integrating sphere, the integrating sphere was purged with dry argon to maintain an inert atmosphere.

2. Device fabrication and characterization

The layers of ITO/1,1-bis[4-[*N*,*N*'-di(p-tolyl)amino]-phenyl]-cyclohexane (TAPC, 30 nm)/ 4,4',4''-Tris(carbaz;Tris(4-(9*H*-carbazol-9-yl) (TCTA, 15 nm)/emitter: 4,4′-bis(*N*-carbazolyl)-1,1′-biphenyl (CBP, 5 wt%, 15 nm)/1,3,5-tri[(3-pyridyl)phen-3-yl]benzene (TmPyPB, 65 nm)/LiF (1 nm)/Al (100 nm) were successively deposited on the pre-cleaned ITO glass substrates at a pressure of less than 10^{-4} Torr. The active area of devices was 2 mm × 2 mm. The EL spectra of devices were measured by fiber optic spectrometer (Ocean Optics USB 2000) in the normal direction. The *J-V-L* curves were investigated by a dual-channel Keithley 2614B source measure unit and a PIN-25D silicon photodiode. All the measurements were conducted at room temperature under

ambient condition.

3. Quantum Chemical Calculations

Quantum chemical calculations were performed by the Gaussian 09 program package, density functional theory (DFT) using the B3LYP/6-31G(d) was performed for achieved optimized molecular geometries. Based on the optimized geometric configurations, the dihedral angles of these molecules and the highest occupied molecular orbital (HOMO), as well as the lowest unoccupied molecular orbital (LUMO) were obtained logically. Time-dependent DFT (TD-DFT) calculations were performed at the PBE0/def2-SVP level to obtain the vertical transitions and NTOs（natural transition orbitals）of the singlet and triplet states based on the corresponding S_0 geometries. The SOC (spin-orbit coupling) calculations were performed using the PBE0 functional and the def2-SVP basis set with PySOC package.

4. Lippert-Mataga calculation

The influence of solvent environment on the optical property of our compounds can be understood using the Lippert-Mataga equation, a model that describes the interactions between the solvent and the dipole moment of solute:

$$hc(v_a - v_f) = hc(v_a^0 - v_f^0) + \frac{2(\mu_e - \mu_g)^2}{a^3} f(\varepsilon, n) \quad 1\text{-}1$$

where f is the orientational polarizability of the solvent, $v_a^0 - v_f^0$ corresponds to the Stokes shifts when f is zero, μ_e is the excited state dipole moment, μ_g is the ground-state dipole moment (can be calculated by TDDFT); a is the solvent cavity (Onsager) radius, derived from the Avogadro number (N), molecular weight (M), and density (d = 1.0 g/cm^3); ε and n are the solvent dielectric and the solvent refractive index, respectively; $f(\varepsilon, n)$ and a can be calculated respectively as follows:

$$f(\varepsilon, n) = \frac{\varepsilon - 1}{2\varepsilon + 1} - \frac{n^2 - 1}{2n^2 + 1} \quad 1\text{-}2$$

$$a = (\frac{3M}{4N\pi d})^{1/3} \quad 1\text{-}3$$

According to 1-1, 1-2 and1-3, the μ_e can be calculated as follows:

$$\mu_e = \mu_g + \left\{\frac{hca^3}{2} \times \left[\frac{d(v_a - v_f)}{df(\varepsilon, n)}\right]\right\}^{1/2} \quad 1\text{-}4$$

Equation 1-4 shows that μ_e is proportional to $\left(\frac{d(v_a - v_f)}{df(\varepsilon, n)}\right)^{1/2}$.

5. Experimental section

Synthesis of 4,9-dibromo-6,7-dimethyl-[1,2,5]thiadiazolo[3,4-g]quinoxaline (QBT-2Br)

To a suspension of compound 4,7-dibromobenzo[*c*][1,2,5]thiadiazole-5,6-diamine (2.32 g, 7.16 mmol) in acetic acid (100 mL) was added butane-2,3-dione (0.62 g, 7.26 mmol), and the mixture was then stirred overnight at room temperature. After addition of 500 mL of water, the solid product was filtered and washed with water and methanol to give a yellow granular

solid (2.44 g, yield 91%). ^{1}H NMR (400 MHz, $CDCl_3$), δ (ppm): 2.90 (s, 6H). ^{13}C NMR (100 MHz, $CDCl_3$), δ (ppm): 157.50, 151.73, 138.11, 113.03, 23.30. MALDI-TOF-MS (m/z) calcd for $C_{10}H_6Br_2N_4S$ $[M]^+$: 374.0544. Found: 374.7766.

Synthesis of 4-tert-butyl-N-(4-tert-butylphenyl)-N-(3-methyl-4-(4,4,5,5-tetramethyl-1,3,2-dioxaborolan-2-yl)phenyl)benzenamine (MeTPA-Bpin)

To a mixture of *N*-(4-bromo-3-methylphenyl)-4-*tert*-butyl-N-(4-tert-butylphenyl)-benzenamine (**MeTPA-Br**, 2.79 g, 6.22 mmol), Bis(pinacolato)diboron (3.11 g, 12.43 mol), Pd(dppf)Cl_2 (365 mg) and KOAc (3.71 g, 37.32 mmol) was added a degassed solvent of 1,4-Dioxane (80 mL). The mixture was refluxed for 18 h under the protection of nitrogen. After being cooled to room temperature, the mixture was poured into water (50 mL). It was extracted with DCM (3 × 80 mL) and the combined organic layer was dried over anhydrous magnesium sulfate. The solvent was removed off by rotary evaporation and the residue was passed through a flash silica gel column with petroleum ether (PE)/dichloromethane (DCM) (*v/v*, 3:1) as the eluent to give a white solid (1.36 g, yield 44%). ^{1}H NMR (400 MHz, CD_2Cl_2), δ (ppm): 7.57 (d, *J* = 8.2 Hz, 1H), 7.32 (d, *J* = 11.4 Hz, 4H), 7.03 (d, *J* = 8.7 Hz, 4H), 6.81 (d, *J* = 1.9 Hz, 1H), 6.76 (dd, *J* = 8.2, 2.1 Hz, 1H), 2.43 (s, 3H), 1.35 (s, 18H), 1.35 (s, 12H). MALDI-TOF-MS (m/z) calcd for $C_{33}H_{44}BNO_2$ $[M]^+$: 497.3465. Found: 497.3490.

Synthesis of 2TPA-QBT

To a mixture of **TPA-Bpin** (484 mg, 1.00 mmol), **QBT-2Br** (108 mg, 0.29 mmol), and tetrakis(triphenylphosphine)palladium (Pd$(PPh_3)_4$, 35 mg) was added a degassed mixture of toluene (10 mL), anhydrous ethanol (2 mL) and 2 M potassium carbonate aqueous solution (1.5 mL). The mixture was refluxed for 24 h under the protection of nitrogen. After being cooled to room temperature, the mixture was poured into water (20 mL). It was extracted with DCM (3 × 30 mL) and the combined organic layer was dried over anhydrous magnesium sulfate. The solvent was removed off by rotary evaporation and the residue was passed through a flash silica gel column with petroleum ether (PE)/dichloromethane (DCM) (*v/v*, 2:1) as the eluent to give a deep purple solid (137 mg, yield 51%). ^{1}H NMR (400 MHz, $CDCl_3$), δ (ppm): 7.86 (d, *J* = 8.8 Hz, 4H), 7.34 (d, *J* = 8.6 Hz, 8H), 7.23 (t, *J* = 9.4 Hz, 12H), 2.73 (s, 6H), 1.36 (s, 36H). ^{13}C NMR (100 MHz, $CDCl_3$), δ (ppm): 154.48, 152.80, 148.03, 146.23, 144.82, 137.04, 133.59, 128.27, 127.45, 124.87, 120.46, 34.21, 31.27, 24.01.

Synthesis of 2MeTPA-QBT

The compound **2MeTPA-QBT** was synthesized by the similar synthetic procedure for **2TPA-QBT** with compound **MeTPA-Bpin** (596 mg, 1.20 mmol) and **QBT-2Br** (153 mg, 0.41 mmol) as starting materials and obtained as a deep purple solid with a yield of 41% (160 mg). ^{1}H NMR (400 MHz, CD_2Cl_2), δ (ppm): 7.34 (d, *J* = 8.6 Hz, 8H), 7.22 (dd, *J* = 12.8, 8.4 Hz, 2H), 7.15 (d, *J* = 8.6 Hz, 8H), 7.07 (s, 2H), 6.94 (dd, *J* = 10.9, 2.7 Hz, 2H), 2.68 (s, 6H), 1.93 (s, 3H), 1.91 (s, 3H), 1.32 (s, 36H). ^{13}C NMR (100 MHz, CD_2Cl_2), δ (ppm): 155.23, 153.00, 148.04, 146.28, 145.02, 138.88, 137.74, 132.58, 129.77, 128.58, 126.23, 124.72, 122.63, 118.41, 34.25, 31.22, 23.67, 20.70. MALDI-TOF-MS (m/z) calcd for $C_{64}H_{70}N_6S$ $[M]^+$: 954.5383. Found: 954.5373.

Synthesis of MeTPA-QBT-Br

The compound **MeTPA-QBT-Br** was synthesized by the similar synthetic procedure for **2TPA-QBT** under the controlling of feed ratios with compound **MeTPA-Bpin** (596 mg, 1.20 mmol) and **QBT-2Br** (568 mg, 1.52 mmol) as starting materials and obtained as a black solid with a yield of 59% (470 mg).^{1}H NMR (400 MHz, CD_2Cl_2), δ (ppm): 7.39 – 7.32 (m, 4H), 7.21 –

7.12 (m, 5H), 7.05 (d, *J* = 2.4 Hz, 1H), 6.92 (dd, *J* = 8.4, 2.4 Hz, 1H), 2.82 (s, 3H), 2.73 (s, 3H), 1.87 (s, 3H), 1.33 (s, 18H). ^{13}C NMR (100 MHz, CD_2Cl_2), *δ* (ppm): 157.21, 156.05, 152.46, 148.25, 146.44, 144.77, 138.76, 138.15, 132.37, 130.79, 127.56, 126.23, 124.93, 122.38, 118.15, 111.61, 34.24, 31.17, 23.65, 23.44, 20.59. MALDI-TOF-MS (m/z) calcd for $C_{37}H3_8BrN_5S$ $[M]^+$: 665.2011. Found: 665.1987.

Synthesis of TPA-QBT-MeTPA

The compound **TPA-QBT-MeTPA** was synthesized by the similar synthetic procedure for **MeTPA-QBT-Br** with compound **MeTPA-QBT-Br** (200 mg, 0.30 mmol) and **TPA-Bpin** (194 mg, 0.4 mmol) as starting materials and obtained as a deep purple solid with a yield of 36% (102 mg). ^{1}H NMR (400 MHz, $CDCl_3$), *δ* (ppm): 7.88 (d, *J* = 8.7 Hz, 2H), 7.33 (ddd, *J* = 6.8, 5.8, 2.4 Hz, 9H), 7.26 (s, 1H), 7.25 – 7.19 (m, 9H), 7.15 (d, *J* = 1.8 Hz, 1H), 7.06 (dd, *J* = 8.4, 2.0 Hz, 1H), 2.74 (s, 3H), 2.71 (s, 3H), 1.98 (s, 3H), 1.36 (d, *J* = 1.6 Hz, 36H). ^{13}C NMR (100 MHz, $CDCl_3$), *δ* (ppm): 154.7, 154.53, 153.24, 152.47, 148.12, 147.80, 146.18, 145.73, 145.12, 144.79, 138.63, 138.11, 136.55, 133.62, 132.31, 129.22, 129.05, 128.54, 127.21, 126.13, 125.00, 124.54, 123.27, 120.40, 119.13, 34.36, 31.50, 29.72, 23.94, 21.09. MALDI-TOF-MS (m/z) calcd for $C_{63}H_{68}N_6S$ $[M]^+$: 940.3192. Found: 940.5373.

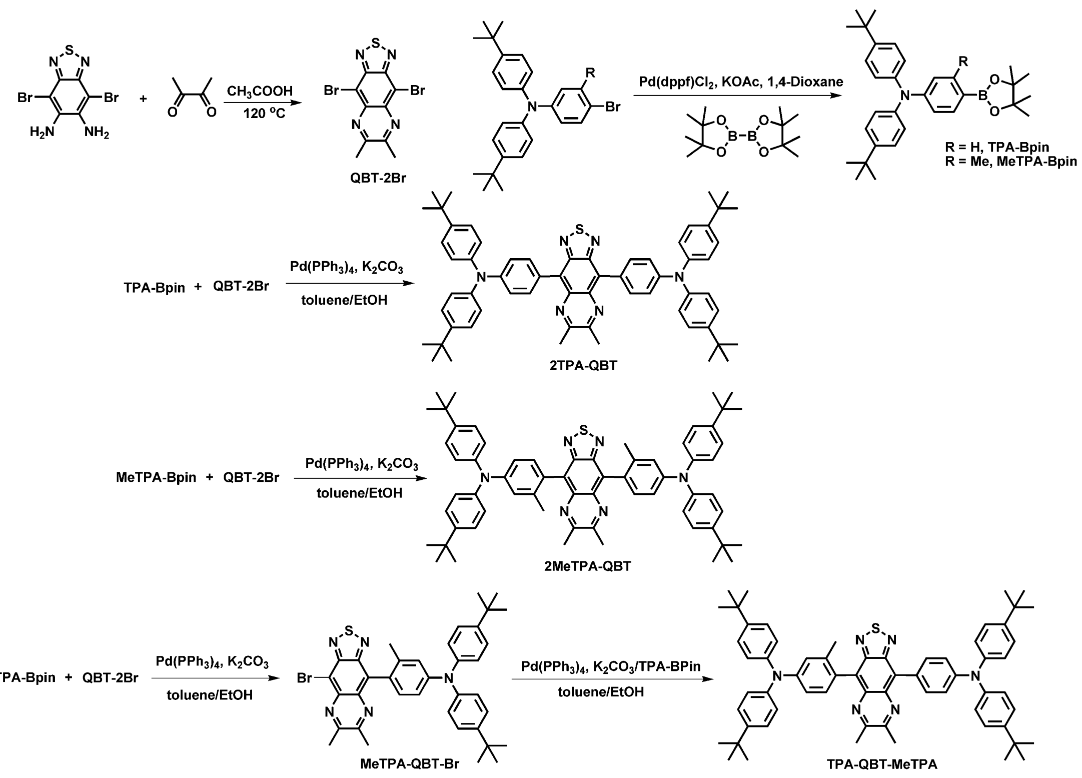

Figure S1. Synthetic routes of **2TPA-QBT, 2MeTPA-QBT** and **TPA-QBT-MeTPA.**

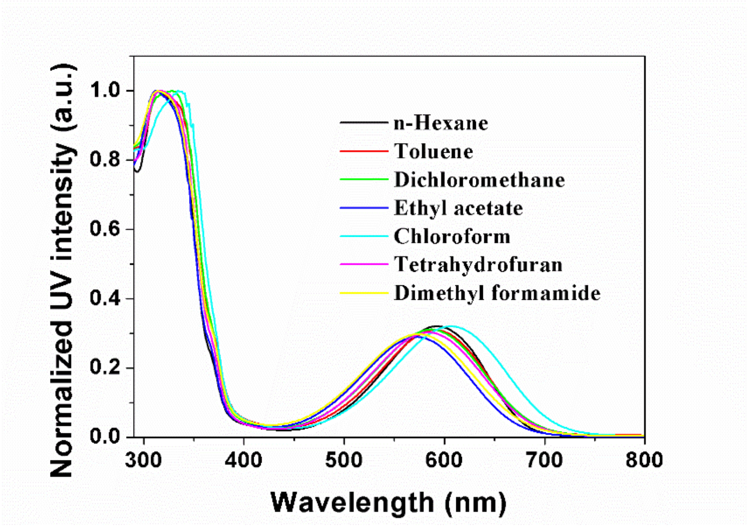

Figure S2. UV-vis absorption spectra of 2TPA-QBT in different solvents

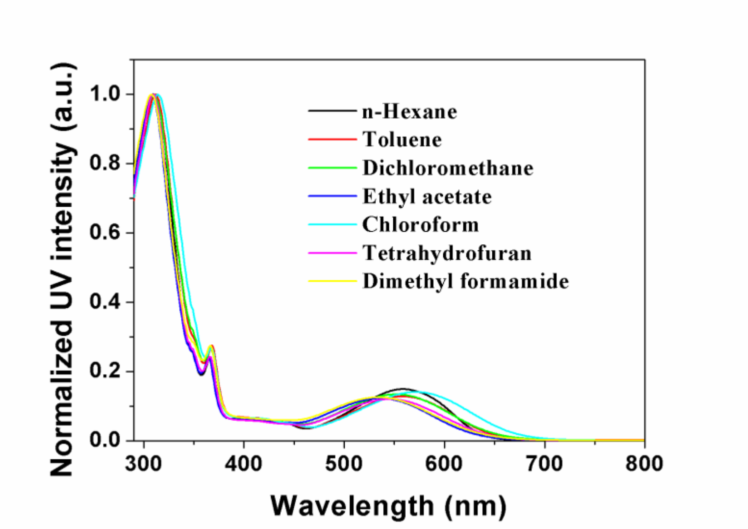

Figure S3. UV-vis absorption spectra of 2MeTPA-QBT in different solvents

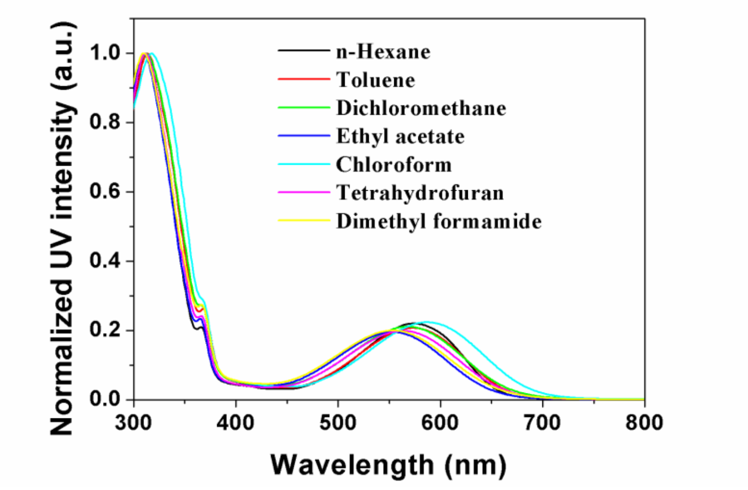

Figure S4. UV-vis absorption spectra of TPA-QBT-MeTPA in different solvents

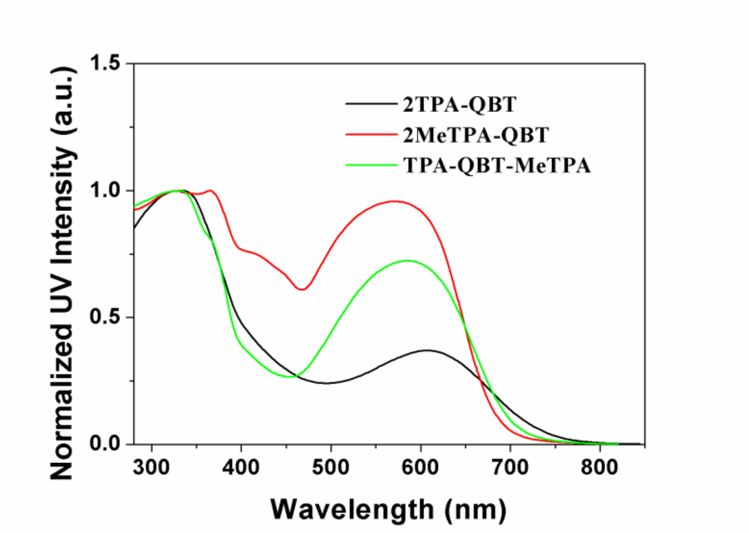

Figure S5. UV–vis absorption spectra of emitters in neat films

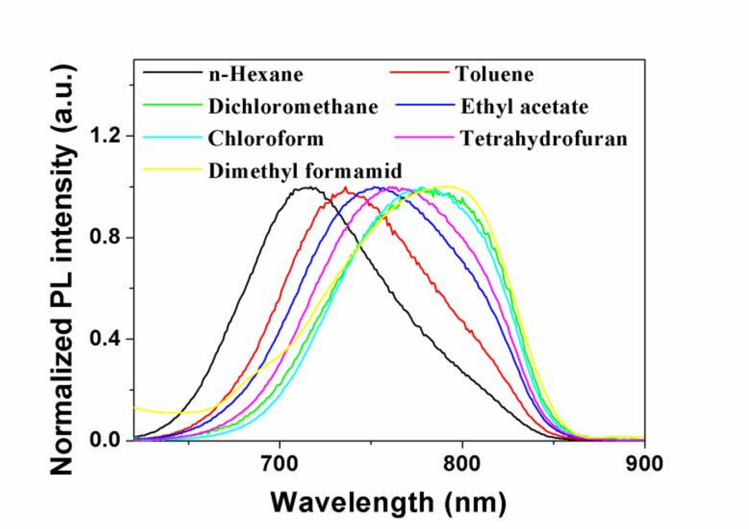

Figure S6. Photoluminescence spectra of 2TPA-QBT in different solvents

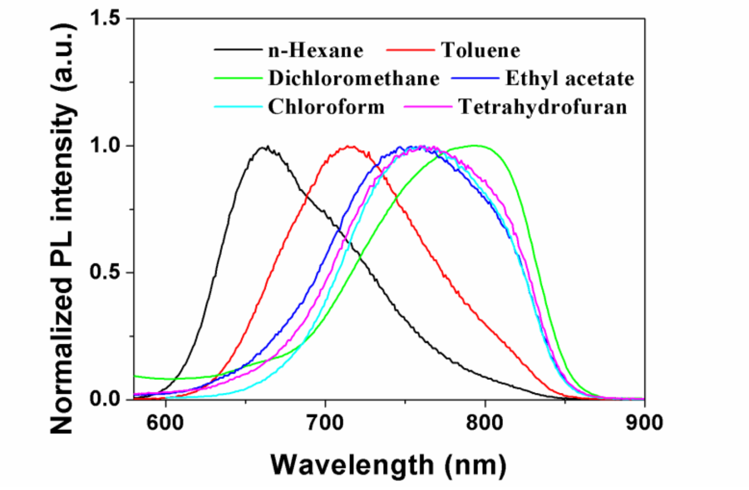

Figure S7. Photoluminescence spectra of 2MeTPA-QBT in different solvents

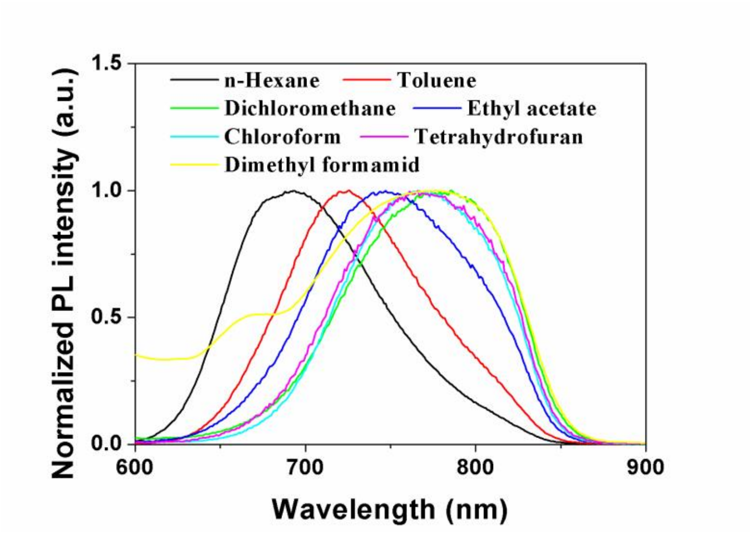

Figure S8. Photoluminescence spectra of TPA-QBT-MeTPA in different solvents

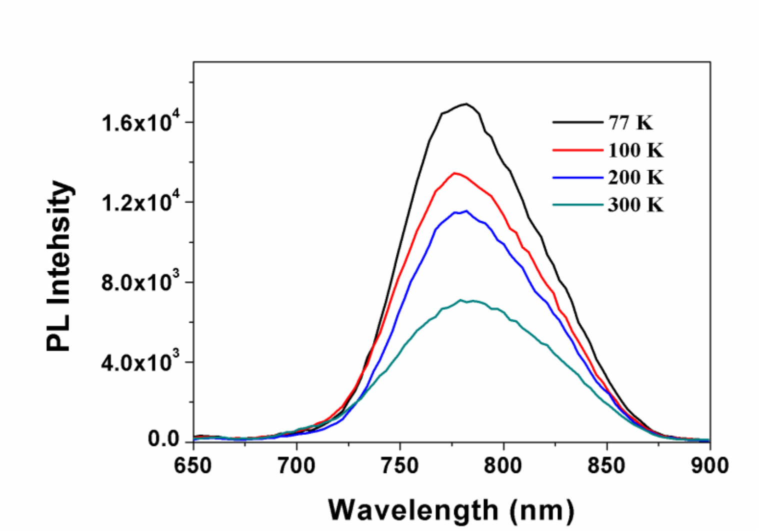

Figure S9. Variable-temperature photoluminescence spectra of 2TPA-QBT in neat films

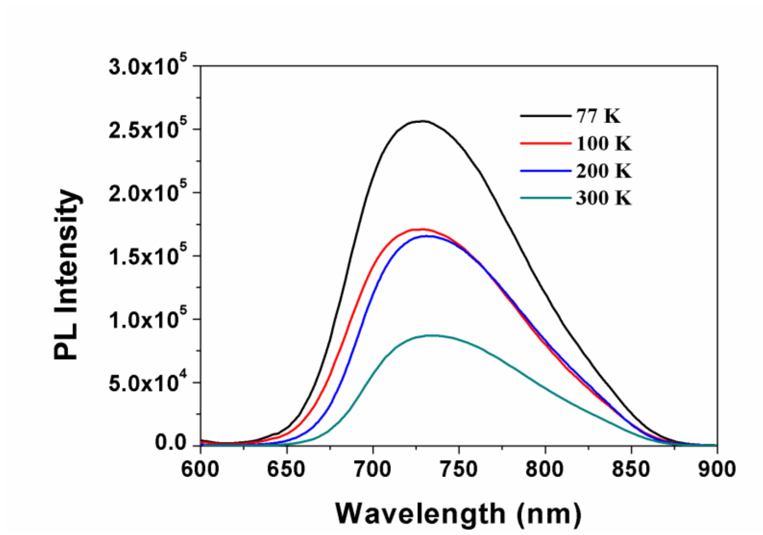

Figure S10. Variable-temperature photoluminescence spectra of 2MeTPA-QBT in neat films

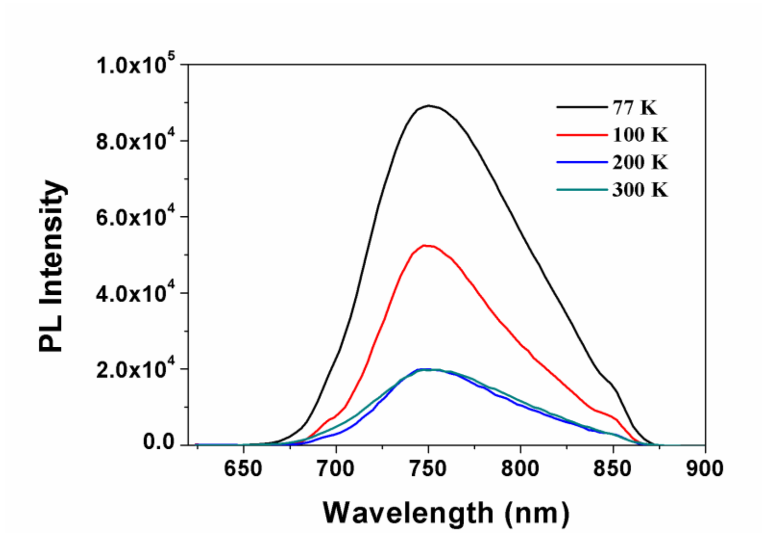

Figure S11. Variable-temperature photoluminescence spectra of TPA-QBT-MeTPA in neat films

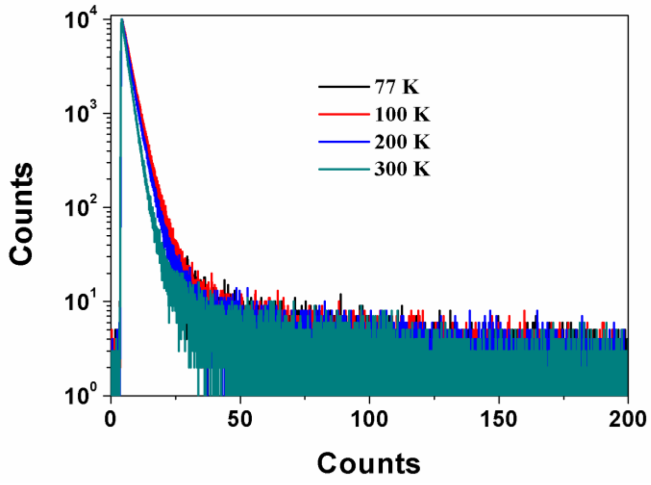

Figure S12. Variable-temperature photoluminescence decay characteristics of TPA-QBT-MeTPA in neat films

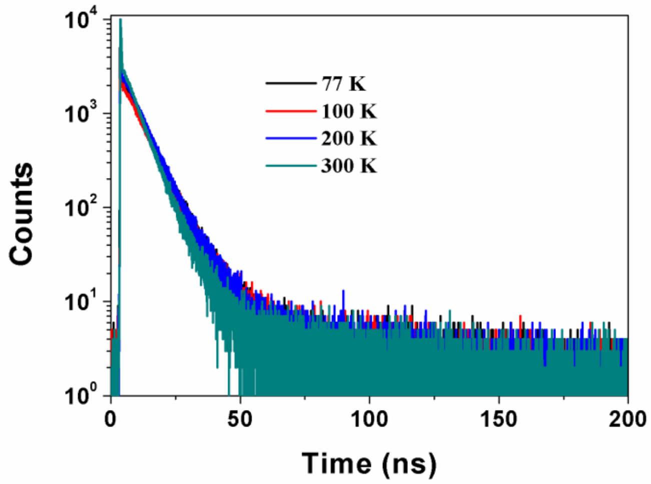

Figure S13. Variable-temperature photoluminescence decay characteristics of 2MeTPA-QBT in neat films

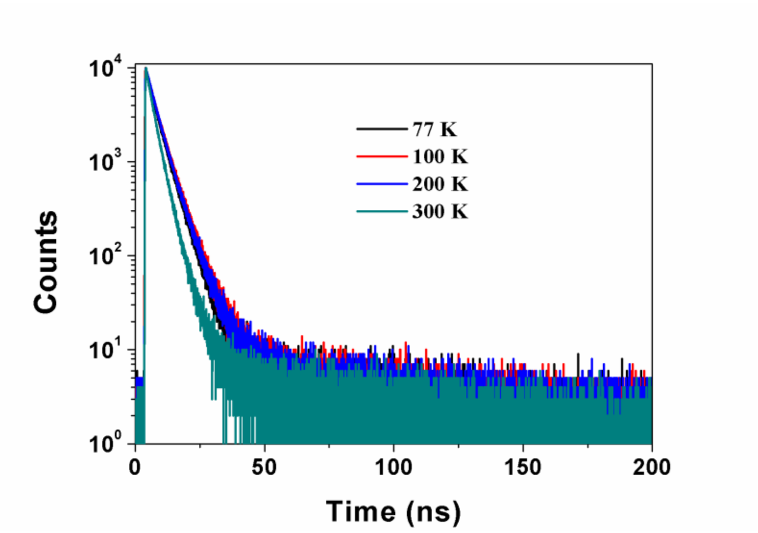

Figure S14. Variable-temperature photoluminescence decay characteristics of TPA-QBT-MeTPA in neat films

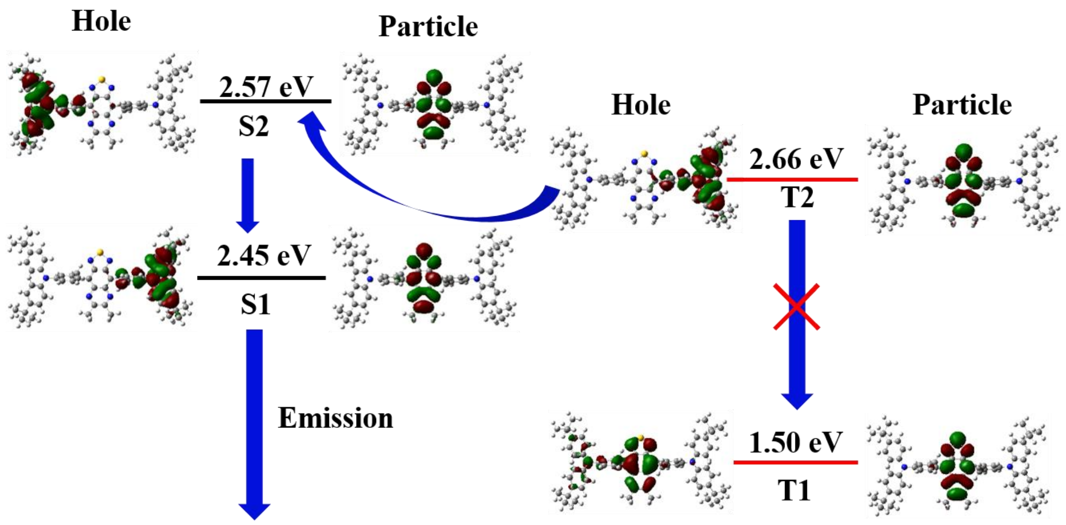

Figure S15. The energy level and the natural transition orbitals of 2TPA-QBT

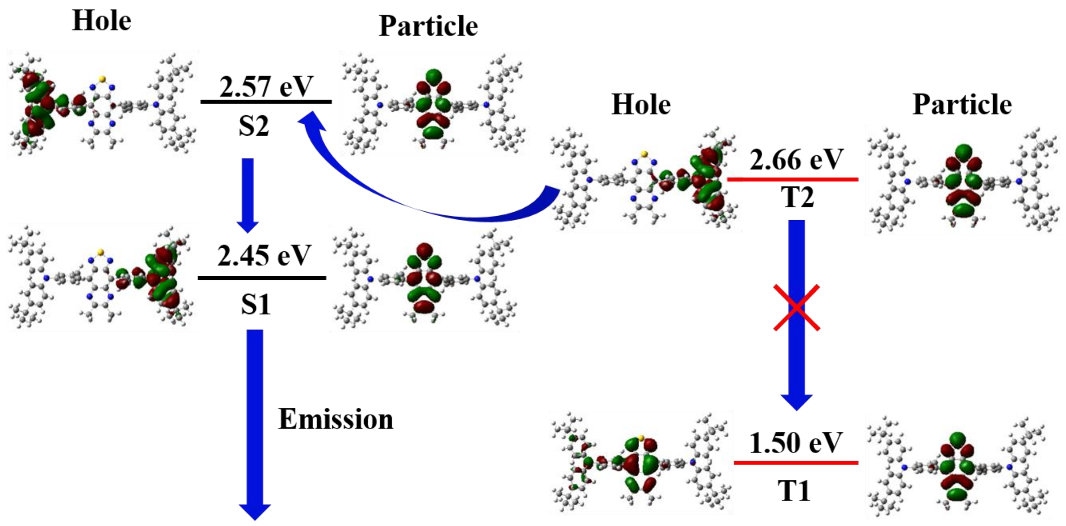

Figure S16. The energy level and the natural transition orbitals of 2MeTPA-QBT

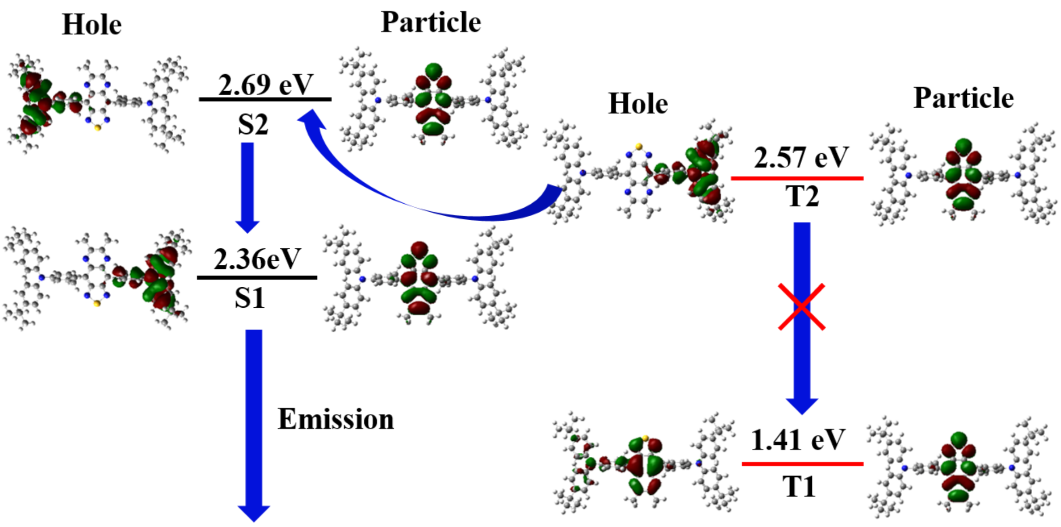

Figure S17. The energy level and the natural transition orbitals of TPA-QBTMeTPA

Table S1. Photophysical properties for the three emitters

Emitters	Solvent	$\lambda_{abs\ sol}$ /nm [a]	$\lambda_{abs\ film}$ /nm [b]	$\lambda_{em\ sol}$ /nm [a]	$\lambda_{em\ film}$ /nm [b]	E_g^{opt}/eV [c]	Φ_F (%) $_{sol}$ [d]	Φ_F (%) $_{film}$ [d]	τ_{sol} /ns [a]	τ_{film} /ns [b]
	n-Hexane	590, 314		714						
	Toluene	589, 314		736						
	Ethyl acetate	571, 312		751						
**2TPA-QBT**	Tetrahydrofuran	583, 315	609	765	780	1.62	26	5	6	3
	Dichloromethane	587, 327		780						
	Chloroform	606, 312		780						
	Dimethyl formamide	571, 334		796						
	n-Hexane	557, 309		662						
	Toluene	557, 312		714						
**2MeTPA-QBT**	Ethyl acetate	527, 309	574	751	732	1.72	38	6	5	6
	Tetrahydrofuran	547, 309		761						
	Dichloromethane	554, 310		797						
	Chloroform	573, 314		760						
	n-Hexane	571, 311		694						
	Toluene	574, 312		724						
	Ethyl acetate	550, 309		746						
**TPA-QBT-MeTPA**	Tetrahydrofuran	561, 311	585	767	748	1.68	34	8	7	3
	Dichloromethane	566, 312		788						
	Chloroform	587, 317		768						
	Dimethyl formamide	553, 310		788						

a) Measured in solution (10^{-5} mol L^{-1}); b) measured in neat film; c) optical band gaps were determined using $E_{g\ film}^{opt} = 1240/\lambda_{onset,\ film}$; d) absolute PL quantum yield measured using an integrating sphere.

^{1}H NMR spectrum of **QBT-2Br**

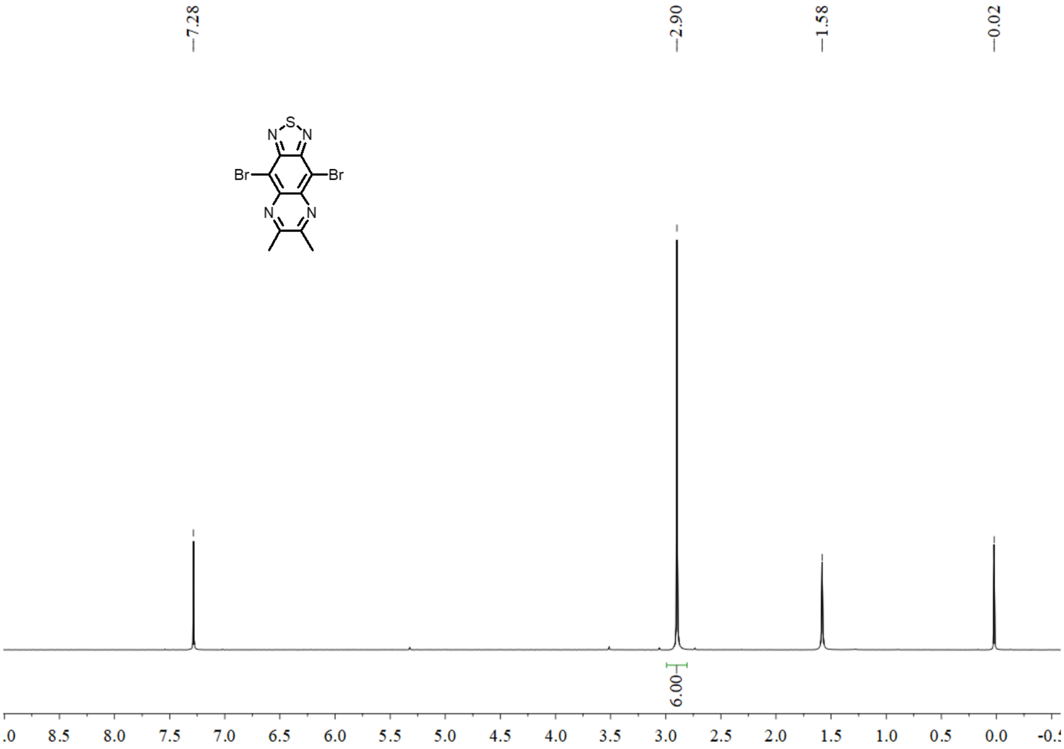

^{13}C NMR spectrum of QBT-2Br

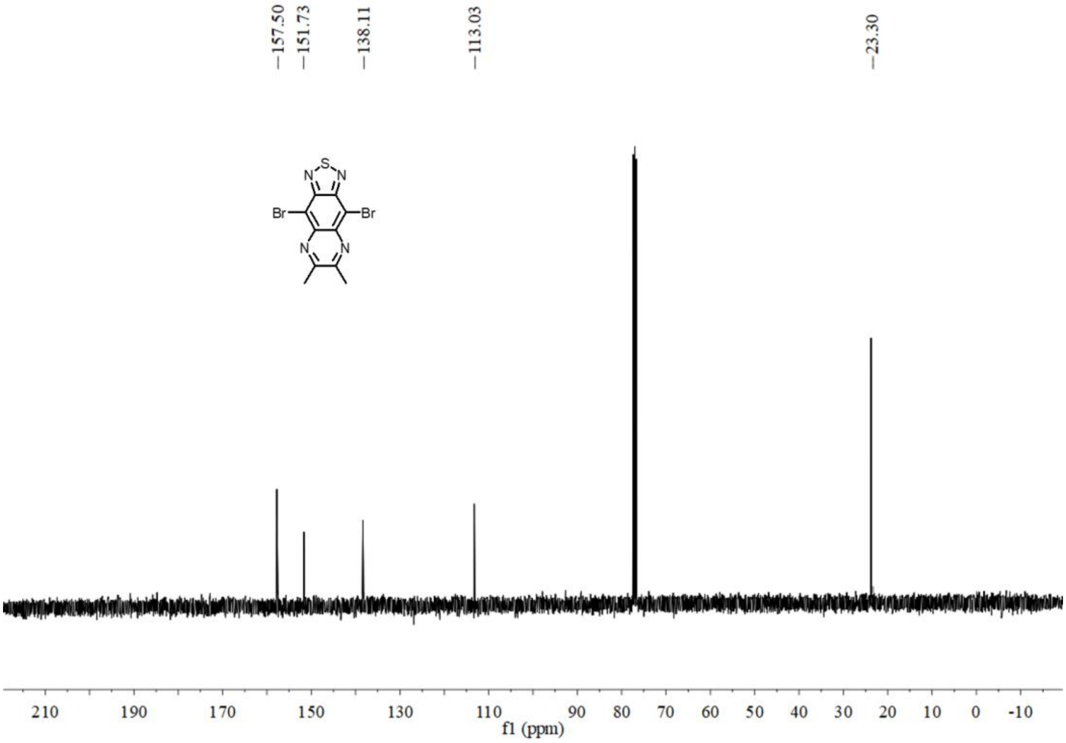

^{1}H NMR spectrum of **MeTPA-Bpin**

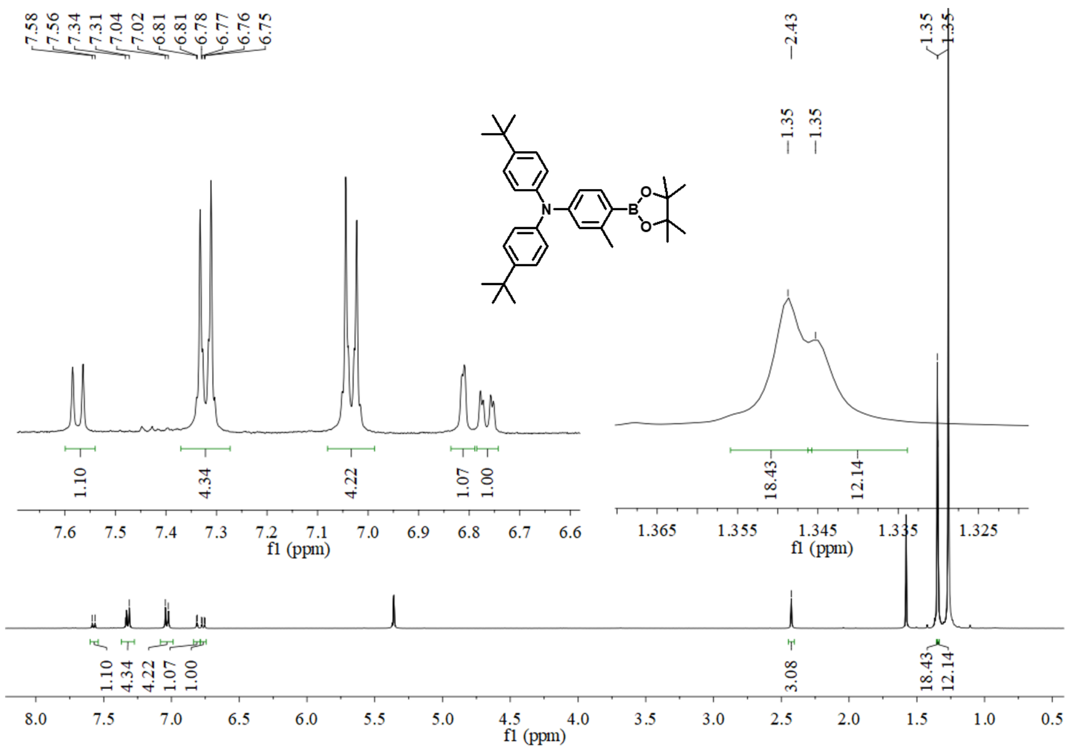

^{1}H NMR spectrum of **MeTPA-QBT-Br**

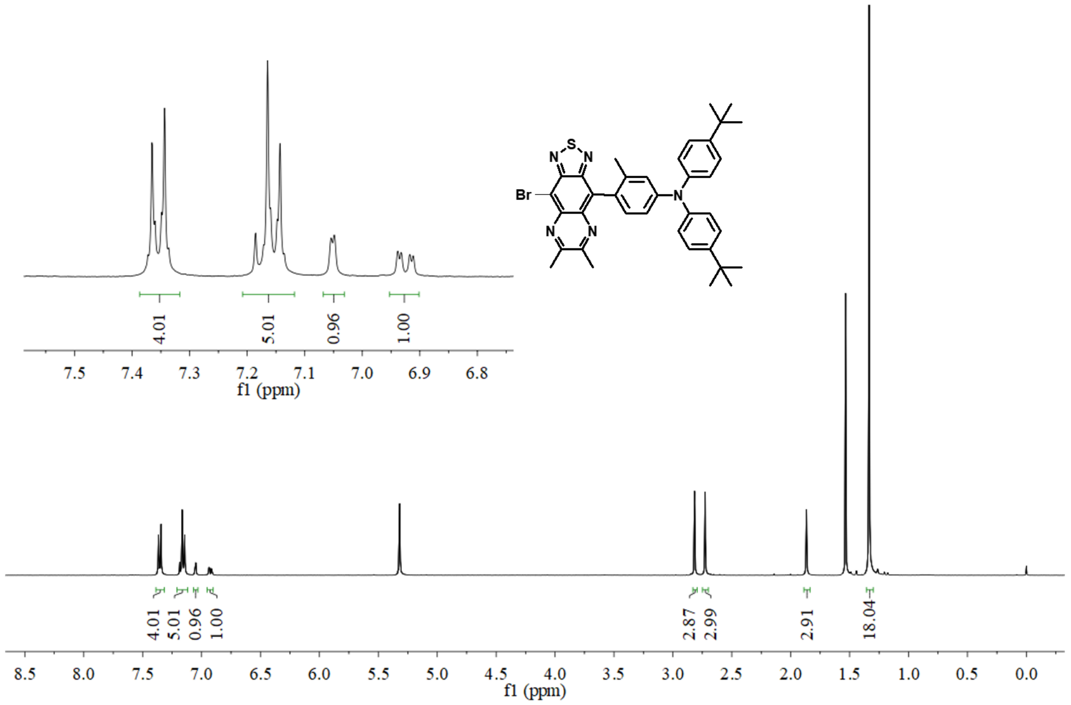

^{13}C NMR spectrum of **MeTPA-QBT-Br**

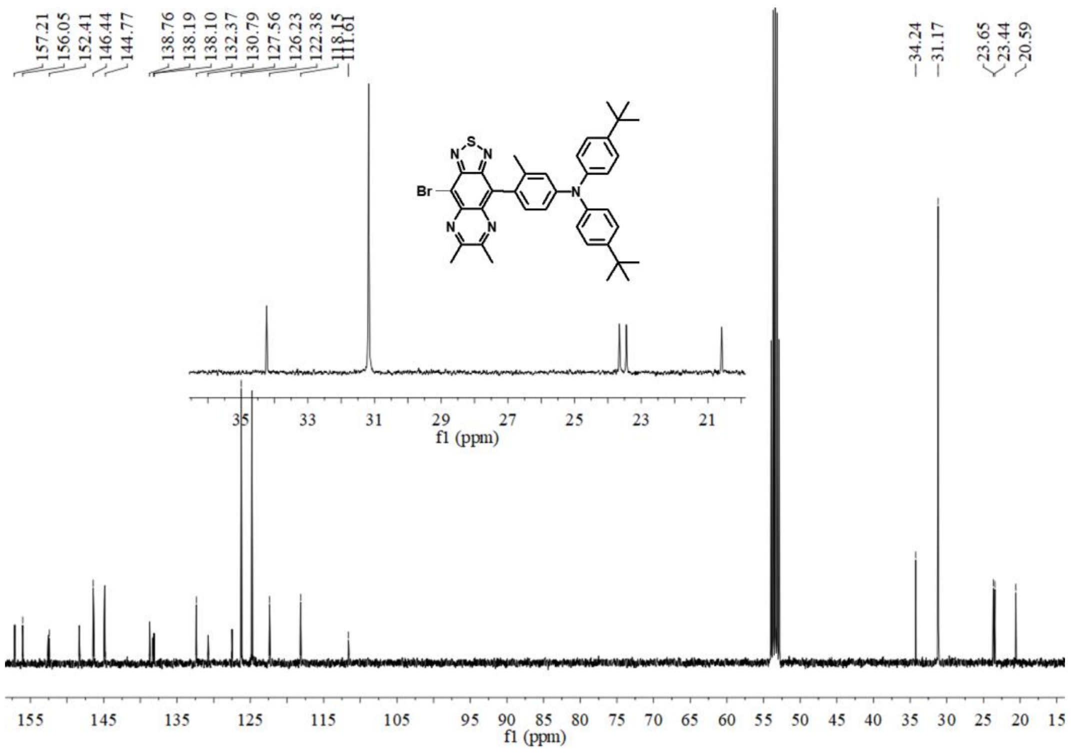

^{1}H NMR spectrum of **2TPA-QBT**

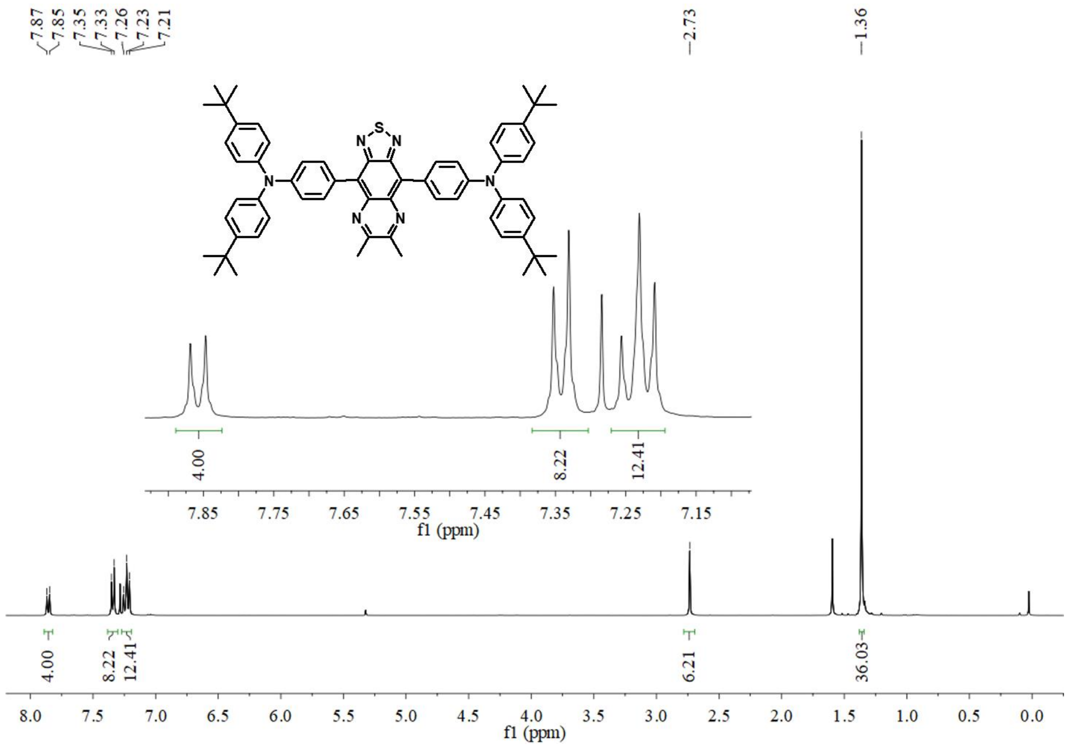

^{13}C NMR spectrum of **2TPA-QBT**

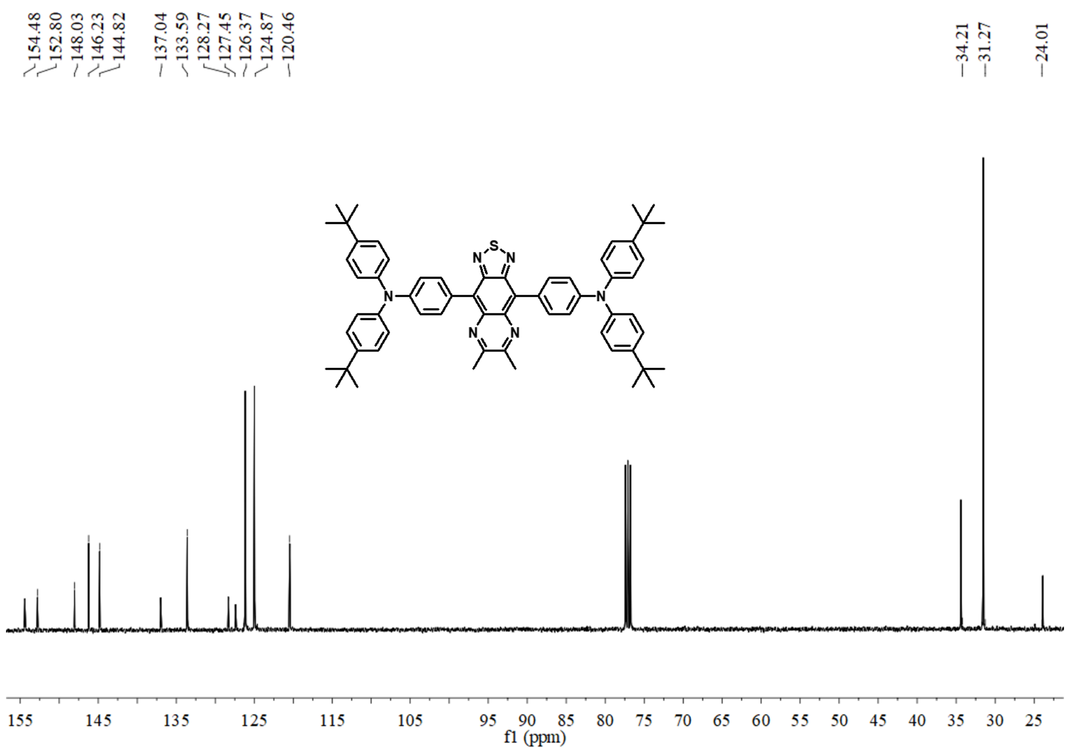

^{1}H NMR spectrum of **2MeTPA-QBT**

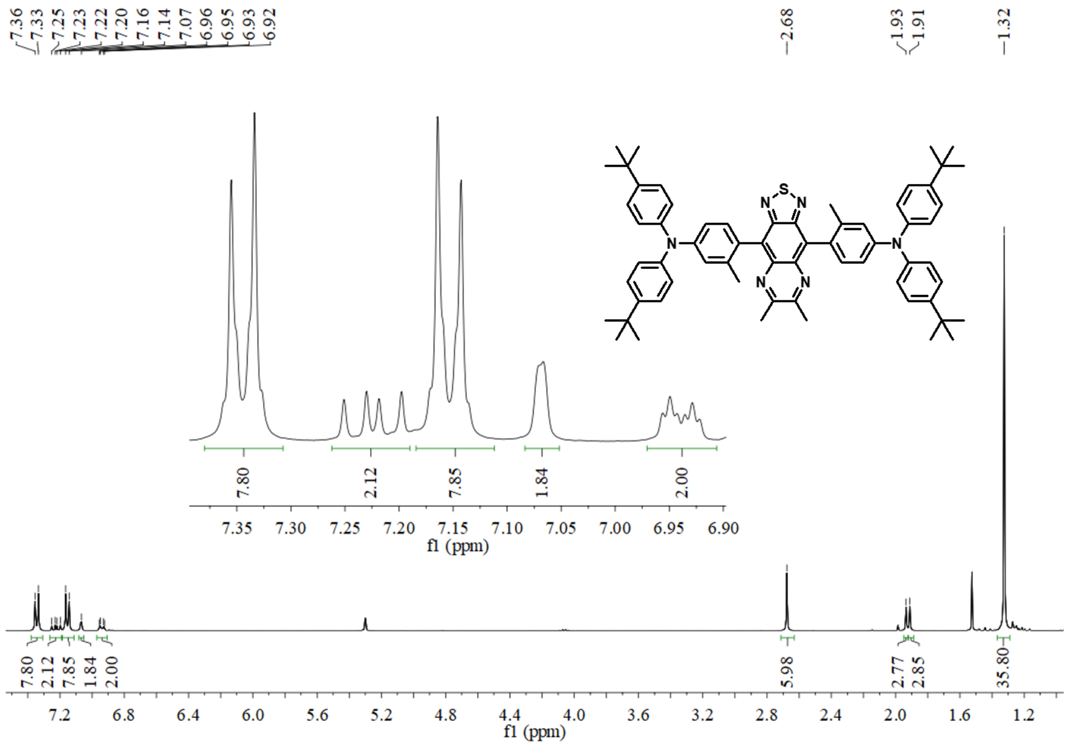

[13] C NMR spectrum of **2MeTPA-QBT**

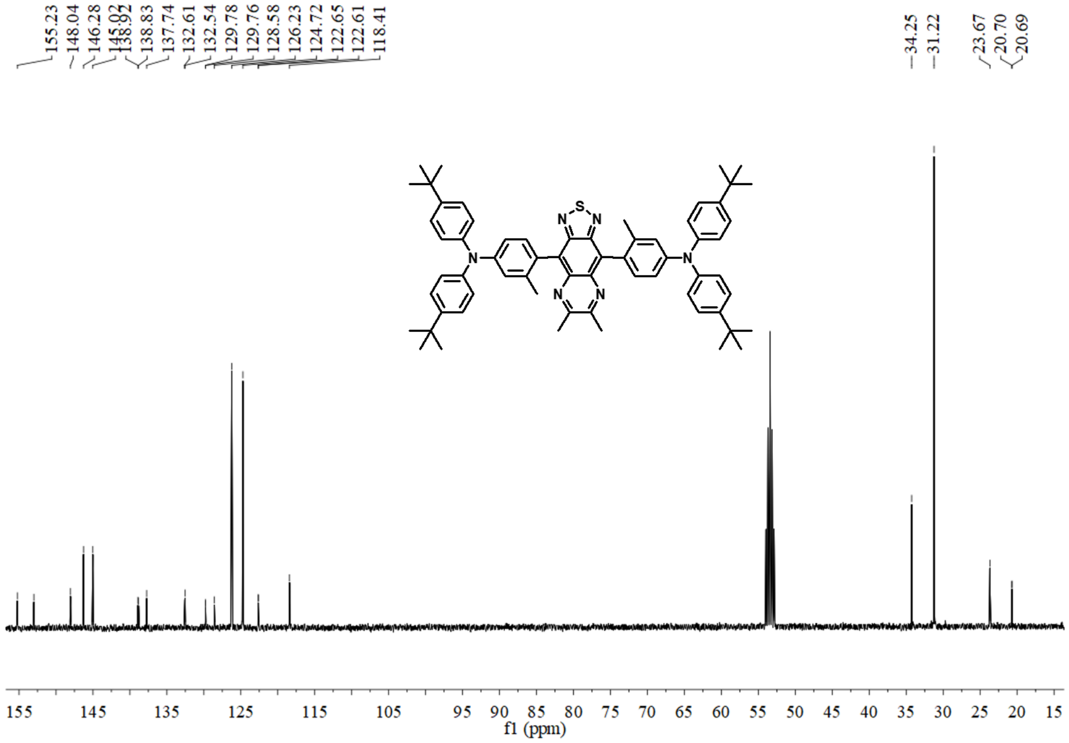

[1] H NMR spectrum of **TPA-QBT-MeTPA**

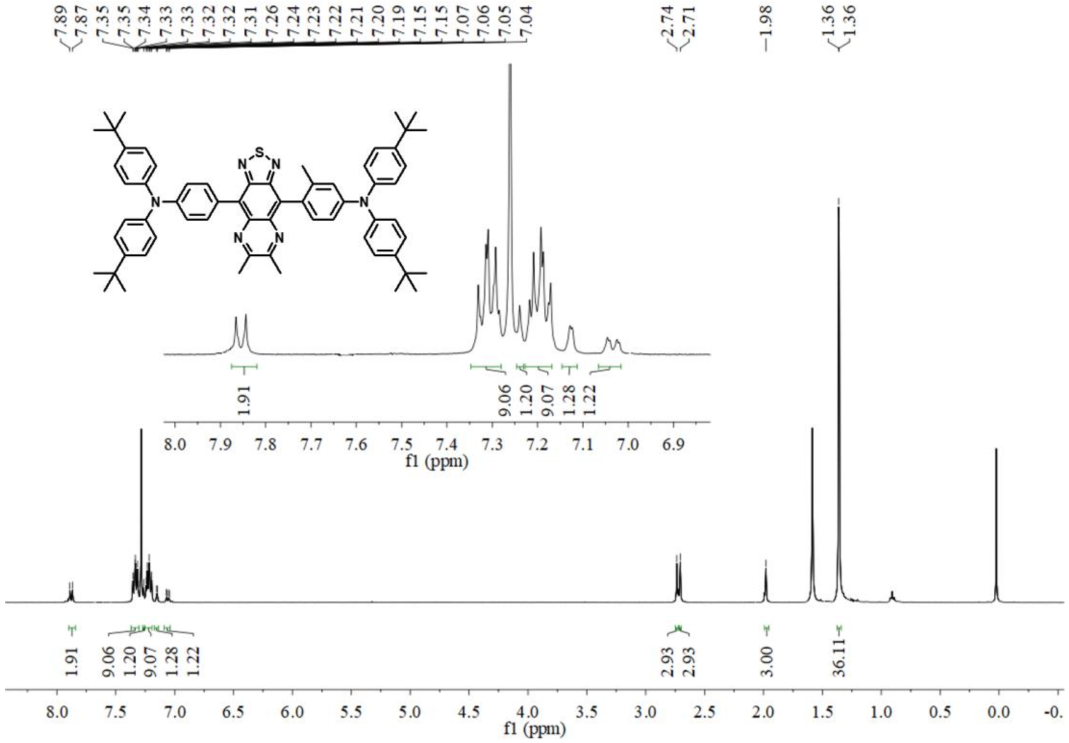

^{13}C NMR spectrum of **TPA-QBT-MeTPA**

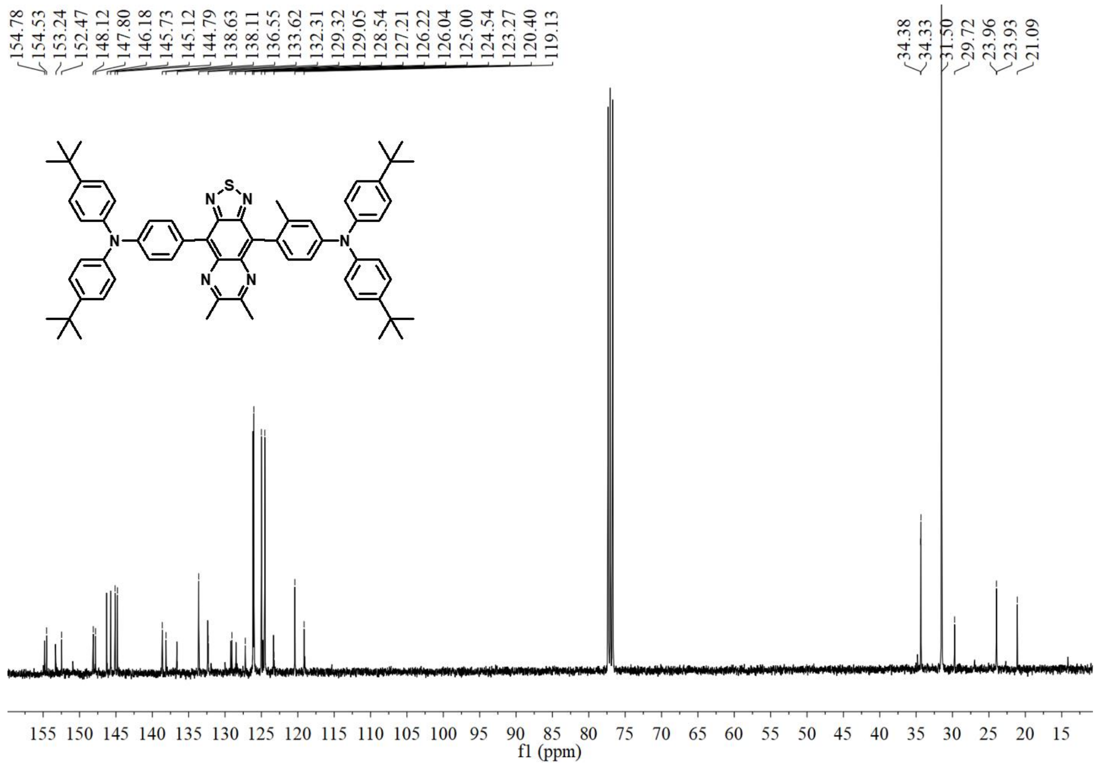

Supplement: Supplementary file 1 [file molecules-26-06386-s001.zip › molecules-1380277-supplementary.pdf]
